# Efficient TALEN‐mediated gene editing in wheat

**DOI:** 10.1111/pbi.13169

**Published:** 2019-06-06

**Authors:** Ming Luo, Hongyu Li, Soma Chakraborty, Robert Morbitzer, Amy Rinaldo, Narayana Upadhyaya, Dhara Bhatt, Smitha Louis, Terese Richardson, Thomas Lahaye, Michael Ayliffe

**Affiliations:** ^1^ CSIRO Agriculture and Food Canberra ACT Australia; ^2^ Eberhard Karls Universität Tübingen ZMBP, Allgemeine Genetik Tübingen Germany; ^3^ The Australian Wine Research Institute Glen Osmond South Australia Australia

**Keywords:** *Triticum*, gene editing, mutation, inheritance, reactivation

Plant genome editing is a major advance in the production of novel plant genotypes. There is, however, only a single previous report of applying this technology to hexaploid wheat using TALEN‐mediated gene editing to produce heritable modifications (Wang *et al*., 2014). Here, we describe highly efficient TALEN editing of a *uidA* transgene and a second endogenous gene, *lr21Ψ,* in bread wheat with efficiencies exceeding most previous TALEN and CRISPR/Cas9 reports in this species.

A TALEN pair (Figure 1a) targeting the *E. coli uidA* gene was co‐transformed into embryos from a wheat cultivar Fielder line segregating for a *Ubi*‐*uidA* transgene. Twelve lines were produced containing both TALENs and at least one copy of *uidA*. T_0_ genomic DNAs of these plants were restricted with *BclI*, as this enzyme cleaves the target site, and the target region was then PCR amplified (Figure 1b). Products were amplified from three of the 12 (25%) DNAs (plants P21, P38 and P45), and Sanger sequencing identified deletions of 3, 13 and 4 bp, respectively, that destroyed the *BclI* site (Figure 1b).

**Figure 1:**
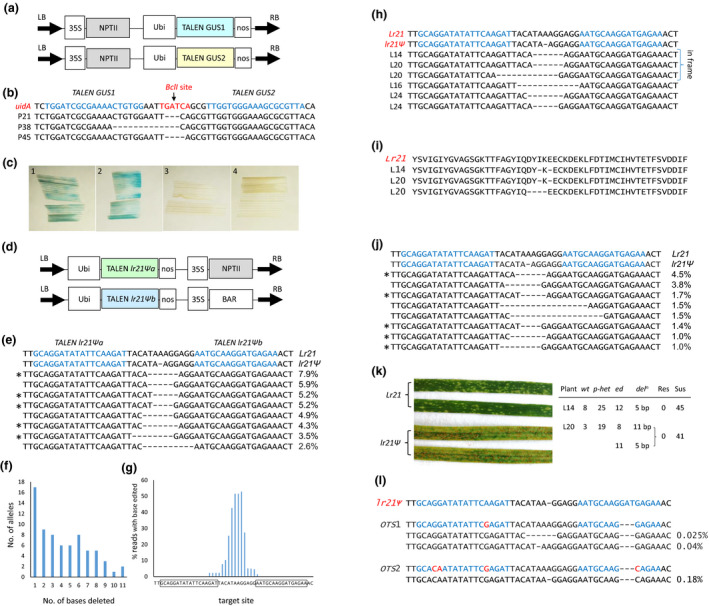
TALEN editing in wheat. (a) Constructs used for TALEN editing of a *uidA* transgene present in Fielder wheat. Each *TALEN,* located on a separate binary vector, was under the regulatory control of maize polyubiquitin promoter (*Ubi*) and *Agrobacterium* nopaline synthase 3′ transcription termination sequences (*nos*). (b) The *uidA* target site sequence with TALEN binding sites shown in blue font. A *BclI* restriction enzyme site is indicated in red font. Edited allele sequences from P21, P38 and P45 are shown. *uidA* sequence corresponds to nucleotides 7799‐7852 of GenBank AB489142.1. Note the actual right‐hand TALEN binding site sequence is the reverse complement as only a single DNA strand is shown for convenience. (c) GUS staining of tissue from T_1_ progeny that contain only edited *uidA* alleles from P21 (panel 2), P38 (panel 3) and P45 (panel 4). Panel 1 shows wild‐type *uidA* staining of unedited P21 sib tissue. (d) TALEN constructs used for editing of *lr21Ψ*. Each TALEN gene was again regulated by maize ubiquitin promoter and *Agrobacterium nos* terminator sequences. (e) The eight most common edited alleles present in *lr21Ψ* T_0_ DNAs with their frequency amongst amplicons from all 40 T_0_ DNAs shown on the right. Sequence shown is equivalent to nucleotide co‐ordinates 1264–1314 of the Fielder *lr21Ψ* sequence (GenBank FJ876295). TALEN binding sites are highlighted in blue. A single nucleotide deletion (A) pre‐exists in the Fielder *lr21Ψ* sequence compared with *Lr21*. Asterisks highlight alleles also present in panel (j). (f) Number of edited *lr21Ψ* alleles, with deletion sizes indicated, that were present in T_0_ DNAs. (g) Deletion frequency of each base at the *lr21Ψ* target site amongst 40 T_0_ DNAs. Boxed sequences on the X‐axis are TALEN binding sites with the reverse complement of the right‐hand TALEN binding site shown. (h) Edited *lr21Ψ* alleles inherited in T_1_ progeny of plants L14, L16, L20 and L24. (i) Predicted peptides encoded by in‐frame edited alleles shown in (h). (j) Eight most common edited amplicon sequences recovered from protoplasts transformed with *lr21Ψ* TALENs. Asterisks highlight alleles also present in T_0_ DNAs (panel e). (k) Screening of TALEN‐edited plants for new leaf rust resistance. The left image shows functional *Lr21* resistance present in wheat accession CS1D5406 when compared with the susceptible cultivar Fielder (*lr21Ψ/ lr21Ψ)*. TALEN‐edited plants L14 and L20 contained restored *lr21Ψ* ORFs as shown in panels (h) and (i). T_1_ progeny of these plants were grown and genotyped for zygosity of alleles with restored ORFs (*wt* = wild *lr21Ψ*,* p‐het* = presumed heterozygous, *ed* = homozygous for the edited allele), and these plants then challenged with *P. triticina*. Heterozygotes are described as presumed as plants producing PCR products with mixed sequencing traces could also be biallelic or chimeric. All seedlings were fully susceptible and indistinguishable to the Fielder control shown on the left. (l) Potential *lr21Ψ TALEN* off‐target sites (*OTS*) amplified from the Fielder wheat genome. *OTS* 1 and *OST2* correspond to annotated wheat genes *TraesCS1B02G002400* and *TraesCS1A02G006700*, respectively. Other off‐target sites are present in the Chinese Spring genome sequence that could not be amplified from Fielder presumably due to sequence polymorphism existing between these two cultivars. TALEN binding sites are highlighted in blue on the *lr21Ψ* and *OTS* sequences. Mismatched nucleotides at the TALEN binding sites of *OTS* sequences are highlighted in red. Immediately beneath each *OTS* are variant sequences identified amongst 40 T_0_ DNAs with frequencies indicated.

T_1_ analysis showed that P38 and P45 were hemizygous for *uidA* and that only edited *uidA* alleles were transmitted to progeny (nine *edited‐uidA*: six null and 10 *edited‐uidA*: 5 null), consistent with an editing event subsequently dominating the majority of germinal tissue in each plant. In contrast, Mendelian inheritance occurred in P21 T_1_ progeny (6 *wt*‐*uidA*: 12 presumed heterozygotes (*p‐hets*): six *edited‐uidA*;* X* ^2^ *P* = 1.0), indicating the parent plant was *uidA/uidA* and a single edited allele predominated in germinal tissue. No GUS staining occurred in P38 and P45 T_1_ plants containing 13‐ and 4‐bp deletion alleles due to loss of *uidA* function, whereas P21 progeny homozygous for a 3‐bp deletion allele showed GUS staining, presumably because the *uidA* ORF remained in‐frame (Figure 1c).

Most gene editing reports have produced targeted gene knockouts. Herein, we attempted to reactivate a pseudogene (*lr21Ψ*) present in Fielder wheat. The functional *Lr21* gene (GenBank AH012974) encodes a nucleotide binding site leucine‐rich repeat protein (NLR) that provides race‐specific resistance to leaf rust disease caused by *Puccinia triticina* (Huang *et al*., 2009). *lr21Ψ* differs to *Lr21* by 3 nonsynonymous SNPS (498 G/D, 854 M/I, 1055 R/S) and a single base deletion that destroys the gene ORF (Huang *et al*., 2009). Previously, a recombinant allele encoding Fielder *lr21Ψ* 5′ regulatory sequences gave functional *Lr21* resistance (Huang et al, 2009). Given this functional expression and near sequence identity between *lr21Ψ* and *Lr21,* we reasoned that restoring the *lr21Ψ* ORF by editing the 1‐bp deletion site may reconstitute a functional resistance gene.

Forty T_0_ plants were produced containing a TALEN pair (Figure 1d) targeting the *lr21Ψ* 1‐bp deletion site (Figure 1e). DNAs from each plant were PCR amplified using primers flanking the *lr21Ψ* target site and amplicons MiSeq sequenced with an average of 2266 ± 800 (standard deviation) reads analysed per sample. Seventy‐three different edited alleles were identified amongst T_0_ amplicons with 71 encoding deletions (1–11 bp; Figure 1f) and two encoding small indels.

Substantial *lr21Ψ* editing occurred with 85% of T_0_ plants (34/40) having between 15% and 100% of amplicons edited. On average, 55% ± 38% (standard deviation) of amplicons from each T_0_ plant were edited. Individual allele frequencies within DNA samples ranged from 0.25% to 98.41% of amplicons, and most alleles (50/73; 68%) were common to at least two plants. The eight most common modified alleles with their respective frequencies are shown (Figure 1e). Nucleotides in the middle of the target site were more commonly deleted than those adjacent to TALEN binding sites (Figure 1g). Target site analysis of 3 Fielder control DNAs showed a very low error rate in this analysis with 0%, 0% and 0.12% of amplicons differing to the wild‐type *lr21Ψ* sequence.

Allele inheritance was investigated in five T_1_ families from plants L14, L16, L20, L24 and L27 using target site PCR amplification and Sanger sequencing. In two T_1_ families, L14‐T_1_ and L16‐T_1_, single edited alleles (Figure 1h) showed Mendelian inheritance (8 *wt*: 25 *p*‐*hets*: 12 *ed*;* X* ^2^ *P* = 0.53 and 7 *wt*: 25 *p*‐*hets*: 9 *ed*;* X* ^2^ *P* = 0.34, respectively). The L14‐T_1_ allele was the most frequent (49.54%) edited sequence amplified from the L14 T_0_ parent, while the L16‐T_1_ allele represented only 8.51% of plant L16 amplicons. Progeny of L20 and L24 inherited two deletion alleles in each family (Figure 1h) although with non‐Mendelian ratios (P20, 3 *wt*: 19 *p*‐*hets*: 11 *ed* with 5‐bp deletion: 8 *ed* with 11‐bp deletion; P24, 0 *wt*: 40 *p*‐*hets*: 6 *ed* with 4‐bp deletion: 2 *ed* with 6‐bp deletion). In both families, these alleles were the most abundant amplicons from the T_0_ parent (L20, 48.27% and 47.21% of amplicons; L24, 49.49% and 47.91%). In contrast, no modified *lr21Ψ* alleles were inherited by progeny of L27 consistent with few amplicons from the parent also being edited (0.46%).

Two edited alleles inherited in T_1_ progeny encoded restored *lr21Ψ* ORFs (Figure 1i). One, present both L14 and L20 T_1_ families, encoded a 5‐bp deletion (Figure 1h) which restored the gene ORF, albeit with the loss of two amino acids (Figure 1i). The second allele, also inherited by L20 progeny, had an 11‐bp deletion (Figure 1h) and encoded an in‐frame ORF with four codons deleted at the editing site (Figure 1h, i). However, *Lr21* resistance to *P. triticina* was not recovered in seedlings homozygous for either of these alleles or amongst 443 T_1_ seedlings from 10 other active *lr21Ψ* TALEN lines which potentially contained additional allelic variants (Figure 1k).

Eight potential *lr21Ψ* TALEN off‐target sites are present in the Chinese Spring wheat genome, and two of these sites, corresponding to genes *TraesCS1B02G002400* and *TraesCS1A02G006700*, were successfully amplified from Fielder DNA. MiSeq sequencing of amplicons from all 40 T_0_ DNAs identified two potential editing events, a 6‐bp and a 1‐bp deletion, at *TraesCS1B02G002400* at low frequency (0.025% and 0.04%) and a single 1‐bp deletion allele at *TraesCS1A02G006700* (0.18% of amplicons; Figure 1l). Little, if any, off‐target editing therefore occurred given these allele frequencies are similar to the background observed in Fielder controls at the *lr21Ψ* site.

It was of interest to compare the editing efficiencies of *lr21Ψ* TALENs in wheat protoplasts with stable transgenics, given protoplast assays are often used for editing studies. Fielder protoplasts were co‐transformed with *Ubi‐YFP* and the *lr21Ψ* TALEN pair. After 2 days of incubation, when 20% of protoplasts showed YFP expression, DNA was extracted and the *lr21Ψ* target site amplified and sequenced (4400 amplicons). A similar, although nonidentical, spectrum of editing events occurred at the *lr21Ψ* target when compared with transgenic wheat plants. Of the 28 alleles amplified from protoplasts, 21 (75%) were also present in wheat T_0_ DNAs. Amongst the eight most frequent edited alleles, five were common to both protoplast and T_0_ DNAs (compare Figure 1e and j). Similar editing events were therefore produced in protoplasts and transgenic plants using this TALEN pair.

These data show highly efficient wheat TALEN editing of a transgene (25%) and an endogenous gene (85%) in T_0_ wheat plants and modified alleles having high heritability. Previously, the wheat *Mlo* gene was TALEN edited with 3.4%–6.0% efficiency in T_0_ plants although with multiple homoeologous loci simultaneously modified in some plants and co‐inherited in T_1_ progeny (Wang *et al*., 2014). Both our and Wang's studies used maize polyubiquitin promoters for TALEN expression, so the large editing efficiency differences between studies suggest variation in TALEN target site accessibility. Alternatively, we used *Agrobacterium* transformation rather than biolistics which may cause significant TALEN expression differences. It is noteworthy that Wang saw higher editing in protoplasts compared with biolistic transgenics.

A variety of stable and transient CRISPR/Cas9 editing approaches have also been used in wheat with T_0_ editing efficiencies usually around 1%–10%, (Howells *et al*., 2018 and references therein; Zhang *et al*., 2019; Kumar *et al*., 2019; Kelliher *et al*., 2019; Okada *et al*., 2019), which is low compared with rice (Zhang *et al*., 2014). While CRISPR/Cas is technically simpler than TALENs and far more amenable to multiplex targeting, not all target sites are efficiently edited in wheat, likely due to sgRNA and genomic target site structural constraints (Yarrington *et al*., 2018; Graf *et al*., 2019). Potentially, this is problematic if an unamenable target site is critical to edit. TALEN editing therefore provides an efficient alternative, without a PAM sequence requirement, and using both platforms will maximize wheat editing opportunities.

While the *lr21Ψ* ORF was successfully restored, resistance gene function was not, possibly due to editing footprints. While this pseudogene reactivation attempt was unsuccessful, others may succeed if less constrained proteins are targeted where editing footprints may be tolerated. Potentially, pseudogene reactivation could be beneficial in introducing a functional allele in breeding programs, which is laborious if wild relatives or unimproved germplasm is the only other available source.

## Conflict of interest

The authors declare that there is no conflict of interest.

## Author contributions

ML, HL, SC and AR undertook experiments; DB, SL and TR produced wheat transgenics; RM and TL designed and produced TALEN constructs; NU and ML undertook bioinformatic analysis; ML and MA designed experiments and wrote the manuscript.

## References

[pbi13169-bib-0001] Graf, R. , Li, X. , Chu, V.T. and Rajewsky, K. (2019) sgRNA sequence motifs blocking efficient CRISPR/Cas9‐mediated gene editing. Cell Reports, 26, 1098–1103. 30699341 10.1016/j.celrep.2019.01.024 PMC6352712

[pbi13169-bib-0002] Howells, R.M. , Craze, M. , Bowden, S. and Wallington, E.J. (2018) Efficient generation of stable, heritable gene edits in wheat using CRISPR/Cas9. BMC Plant Biol. 18, 215. 30285624 10.1186/s12870-018-1433-z PMC6171145

[pbi13169-bib-0003] Huang, L. , Brooks, S. , Li, W. , Fellers, J. , Nelson, J.C. and Gill, B. (2009) Evolution of new disease specificity at a simple resistance locus in crop‐weed complex: reconstitution of the Lr21 gene in wheat. Genetics, 182, 595–602. 19364806 10.1534/genetics.108.099614 PMC2691766

[pbi13169-bib-0004] Kelliher, T. , Starr, D. , Su, X. , Tang, G. , Chen, Z. , Carter, J. , Wittich, P.E. , Dong, S. , Green, J. , Burch, E. , McCuiston, J. , Gu, W. , Sun, Y. , Strebe, T. , Roberts, J. , Bate, N.J. and Que, Q. (2019) One‐step genome editing of an elite crop germplasm during haploid induction. Nature Biotechnol. 37, 287–292. 30833776 10.1038/s41587-019-0038-x

[pbi13169-bib-0005] Kumar, R. , Kaur, A. , Pandey, A. , Mamrutha, H.M. and Singh, G.P. (2019) CRISPR‐based genome editing in wheat: a comprehensive review and future prospects. Mol. Biol. Rep. 10.1007/s11033-019-04761-3. 30941642

[pbi13169-bib-0006] Okada, A. , Arndell, T. , Borisjuk, N. , Sharama, N. , Watson‐Haigh, N.S. , Tucker, E.J. , Baumann, U. , Langridge, P. and Whitford, R. (2019) CRISPR/Cas9‐mediated knockout of Ms1 enables the rapid generation of male‐sterile hexaploid wheat lines for use in hybrid seed production. Plant Biotechnol. J. 10.1111/pbi.13106. PMC6737020 30839150

[pbi13169-bib-0007] Wang, Y. , Cheng, X. , Shan, Q. , Zhang, Y. , Liu, J. , Gao, C. and Qiu, J.‐L. (2014) Simultaneous editing of three homoeoalleles in hexaploid bread wheat confers heritable resistance to powdery mildew. Nat. Biotech. 32, 947–952. 10.1038/nbt.2969 25038773

[pbi13169-bib-0008] Yarrington, R.M. , Verma, S. , Schwartz, S. , Trautman, J.K. and Carroll, D. (2018) Nucleosomes inhibit target cleavage by CRISPR‐Cas9 in vivo. Proc. Natl. Acad. Sci. 115, 9351–9358. 30201707 10.1073/pnas.1810062115 PMC6156633

[pbi13169-bib-0009] Zhang, Z. , Hua, L. , Gupta, A. , Tricoli, D. , Edwards, K.J. , Yang, B. and Li, W. (2019) Development of an Agrobacterium‐delivered CRISPR/Cas9 system for wheat genome editing. Plant Biotechnol. J. 10.1111/pbi.13088. PMC6662106 30706614

[pbi13169-bib-0010] Zhang, H. , Zhang, Z. , Wei, P. , Zhang, B. , Gou, F. , Fen, Z. , Mao, Y. , Yang, L. , Zhang, H. , Xu, N. and Zhu, J.‐K. (2014) The CRISPR/Cas9 system produces specific and homozygous targeted gene editing in rice in one generation. Plant Biotechnol. J. 12, 797–807. 24854982 10.1111/pbi.12200

